# Spatio-Temporal Evolution and Influencing Factors of Ecological Well-Being Performance from the Perspective of Strong Sustainability: A Case Study of the Three Gorges Reservoir Area, China

**DOI:** 10.3390/ijerph20031810

**Published:** 2023-01-18

**Authors:** Zhicheng Lai, Lei Li, Zhuomin Tao, Tao Li, Xiaoting Shi, Jialing Li, Xin Li

**Affiliations:** 1 School of Geography, Nanjing Normal University, Nanjing 210023, China; 2 Jiangsu Center for Collaborative Innovation in Geographical Information Resource Development and Application, Nanjing 210023, China

**Keywords:** ecological well-being performance, strong sustainability, Three Gorges Reservoir Area, network SEBM model, Geodetector, PCA-DEA, spatio-temporal evolution, influencing factors

## Abstract

The concept of strong sustainability suggests that natural capital is irreplaceable and emphasizes that human natural consumption cannot exceed the carrying capacity of the ecological environment. In the realistic context of tightening resource constraints and ecological degradation, how to explore the optimal economic and well-being output under certain ecological constraints has become an important topic in sustainability research. Ecological well-being performance (EWP) can comprehensively reflect the efficiency of natural resources and ecological inputs into the well-being level and has become an important tool for sustainable development research. Based on strong sustainability, this paper constructs an EWP evaluation index system. It decomposes EWP into two stages: ecological economy and economic well-being, which opens the “black box” of the ecological well-being conversion process. PCA-DEA, the network super-efficiency Epsilon-based measure (Network SEBM) model, and Geodetector are used to dynamically measure the EWP in the Three Gorges Reservoir Area (TGRA) from 2010 to 2020 and analyze its spatial and temporal characteristics and influencing factors. The results show that (1) temporally, the EWP in the TGRA shows an increasing trend from 2010 to 2020, but the overall level is low. Spatially, it shows a high pattern in the east and low in the west, and spatial differences are gradually decreasing; (2) ecological economic efficiency is significantly lower than economic well-being efficiency, and ecological economic efficiency is the main reason limiting the improvement in EWP in the TGRA. The ecological well-being situation of the TGRA is not optimistic; (3) there is an overall problem of excessive ecological input and insufficient per-capita GDP and well-being output in the TGRA, and decisions should be made according to local conditions; (4) the level of economic development has an EWP level that plays a dominant role and also has a greater relationship with the policy system, socioeconomic conditions, and natural environment.

## 1. Introduction

Since the concept of sustainable development was introduced in 1987 [1], two research paradigms have been developed: the weak-sustainability research paradigm proposed by neoclassical economics and the strong-sustainability research paradigm proposed by sustainability economics [2]. Weak sustainability (WS) assumes that the economy can grow indefinitely, independent of ecosystem constraints, and emphasizes the substitutability of natural capital [3]. Strong sustainability (SS), on the other hand, emphasizes the fact that human natural consumption cannot exceed the carrying capacity of the ecological environment; otherwise, economic growth and the improvement in human well-being will be constrained by the absolute scarcity of natural capital [4,5]. SS imposes additional requirements on the consumption of natural capital based on WS and requires maintaining the stock level of natural capital between generations, which is highly compatible with the construction of ecological civilization in China and is important for promoting sustainable development [4,5]. In 2012, at the Rio+20 Global Conference on Sustainable Development, strong sustainability was officially recognized as a new green economic paradigm. Strong sustainability requires that human economic and social development must respect the boundaries and natural limits of the earth [6].

Since 1978, China has made remarkable socio-economic achievements [7]. However, at the same time, it has also generated issues such as resource depletion, environmental pollution, and ecological degradation [8]; additionally, the contradiction between economic development, environmental protection, and well-being enhancement has intensified [9], which to a certain extent has hindered sustainable socioeconomic development and affected people’s well-being [10]. In the context that human society has transitioned from an “empty world” to a “full world” and the scarcity of natural capital has become a major constraint to human development [11,12], from the concept of SS, exploring the optimal economic and well-being output under certain ecological constraints has become a hot academic issue that needs to be addressed [13]. The concept of ecological well-being performance (EWP), first proposed by Daly in 1974, has gradually entered the academic field [14]; it is an important indicator reflecting the coordination between economy, society, and ecology, belongs to the category of SS concepts, is in line with the general requirements of ecological civilization construction in China, and has been widely used in regional sustainable development research.

Compared with the previous literature, this study focuses on the EWP of the Three Gorges Reservoir Area (TGRA) in China, because the TGRA is an artificial reservoir area that combines ethnic areas, ecologically fragile areas, and concentrated contiguous poverty areas [15,16]; additionally, it is an important ecological barrier area in the Yangtze River basin with an important strategic position. Its ecological security status is extremely important to the ecological security pattern of the whole of China [16]. In addition, the region is facing long-term problems of ecological structure imbalance, insufficient land for construction, and severe soil erosion due to the Three Gorges Dam Project and geographic climate [17]. Given these challenges, this paper scientifically measures and evaluates the EWP of the TGRA from 2010 to 2020, analyzes the spatial and temporal differences in the EWP within the TGRA, and provides policy suggestions for its future development based on the SS research paradigm. This study, which is conducive to promoting the coordination of the economy, society, and environment within the TGRA, encourages the sustainable development of the region and the enhancement of people’s well-being and provides a reference basis for promoting the construction of ecological civilization and high-quality development in China’s Yangtze River Economic Belt.

## 2. Literature Review

### 2.1. Strong Sustainability

At present, sustainable development has been widely recognized and supported, but there has been controversy about the definition, connotation, and assessment of sustainable development. As the controversy has developed, two views of strong sustainability and weak sustainability have gradually emerged [18], with the most important point of disagreement between the two being whether to recognize the substitutability of natural and manufactured capital [2,19,20]. WS, as proposed by neoclassical economists, assumes that natural and manufactured capital are substitutable and that there is no essential difference between the types of well-being they produce [20,21,22]. They, therefore, assume that economic, social, and environmental systems are juxtaposed and that as long as the combined wealth in the sense of the sum of the three is growing, it is sustainable [18]. This implies that economic development does not need to consider the ecological limits and the impact of natural capital depletion on future social well-being levels. However, there are limits to the substitution between natural capital and other capital, and when certain critical natural capital is depleted, it will affect human survival and well-being enhancement and constrain sustainable development [21,23].

As human exploitation of nature deepens and the scarcity of natural capital becomes a major limiting factor for human prosperity, the concept of strong sustainability proposed by sustainable economists is gradually being accepted by the academic community [24,25]. SS argues that there are qualitative differences between natural and manufactured capital [21], that manufactured capital is not a complete substitute for natural capital [2], and that natural capital, manufactured capital, and other forms of capital (e.g., human capital and social capital) create human well-being that is complementary, not identical [26]. At the same time, certain critical natural capital makes a unique contribution to human well-being [21], and thus SS argues that only integrated capital growth with non-decreasing critical natural capital is sustainable. At the same time, SS also emphasizes intergenerational equity, requiring the preservation of equal amounts of natural resources for current and future generations.

On the other hand, the concept of strong sustainability defines that natural capital is fundamental for human well-being and economic growth [20]. Human consumption of nature must not exceed the carrying capacity of the ecological environment; otherwise, the absolutely scarce natural capital will continue to decrease, thus further constraining human well-being and economic growth [18]. Therefore, based on the research paradigm of strong sustainability, “sustainable development” can be considered as “achieving a higher level of well-being within the carrying capacity of the ecological environment”, i.e., bringing about the greatest well-being enhancement with the least ecological consumption under certain ecological constraints [18]. The characterization of strong sustainability also needs to be carried out in two dimensions: well-being level and natural consumption. In the previous literature, a large number of scholars have explored the characterization methods of strong sustainability, such as using GDP/GNP/ISEW/CPI/HDI/SDGs to characterize well-being level [27] and the ecological footprint (EF)/environmental performance index/green city index to characterize natural consumption [28,29].

In terms of applications of SS, Uehara and Mineo found that Japan’s integrated coastal zone should be managed from a strong-sustainability perspective by defining domain values [30]. Shang et al., investigated 30 years of data from Mongolia and found that weak sustainability is not sustainable and recommended a strong-sustainability approach [31]. Shmelev and Rodrígues-Labajos took a strong-sustainability perspective and analyzed the performance of 15 European countries, based on six indicators defined as thresholds by the European Commission [32]. In addition, Pinto et al. developed a model to apply strong sustainability to the sustainability assessment of manufacturing companies [20]. Pelenc and Ballet analyzed the relationship between human well-being and key natural capital to demonstrate the implementation of human development projects from a strong-sustainability perspective [19]. Jain and Jain based on strong sustainability, the sustainable human development index (SHDI) was constructed and its validity was demonstrated through a comparative analysis with the HDI [33].

### 2.2. Ecological Well-being Performance

Daly was the first to study EWP, which he considered the degree of human well-being enhancement per unit of ecological resource input, and defined it as “service/throughput” [14]. However, the use and promotion of the concept of EWP were limited by the lack of indicators to quantify the number of services and throughput precisely. Rees further proposed the concept of ecological footprint (EF) based on Daly’s work, which turned the ecological inputs in EWP into measurable indicators and promoted the development of EWP [34]. Later, Zhu et al. quantified it as the increase in human well-being per unit of ecological consumption, using the ratio of the human development index (HDI) to ecological footprint (EF) for empirical evidence [35]. EWP reflects the efficiency of economic and well-being output under certain ecological constraints and belongs to the category of the strong sustainability concept. Improving EWP is important for achieving regional sustainable development and improving human well-being [36].

In recent years, research on EWP has focused on the following aspects. First, in the measurement and evaluation of EWP, the ratio and the modeling method are mainly used. The ratio method is used to measure EWP by the ratio of HDI and EF [35,37,38]. Since HDI and EF can be applied to various geographical scales (global, regional, national, urban, and community) [39], the ratio method is widely used, which greatly broadens the scope of EWP use. However, the HDI in the ratio method ignores the environmental dimension emphasized by the Sustainable Development Goals 2030 (SDGs) [40]. It cannot comprehensively and objectively reflect the level of EWP under multiple inputs and outputs. Therefore, modeling methods based on the multi-input–multi-output perspective were born, including the stochastic frontier approach (SFA) and data envelopment analysis (DEA) methods. For example, Xiao et al. and He et al. measured the EWP of China and Jiangsu Province, respectively, by the improved SFA model [27,41], while Wang et al. and Bian et al. evaluated the EWP of Poyang Lake and 285 cities in China, respectively, using the improved DEA model (SBM model) [42,43]. However, the modeling approach has some drawbacks, such as multiple input and output indicators that may lead to problems in data acquisition and processing and limit its use to compare multiple countries and regions [36].

In addition, impact factors are a popular area of EWP research and have been extensively studied by scholars. Most studies have identified economic growth as an important factor influencing EWP, and some useful conclusions have been made about the relationship between EWP and economic growth [44]. He et al. found that there is a significant negative spillover effect of the level of economic development on EWP in Jiangsu Province [41]. Dietz et al. found that GDP per capita shows a U-shaped relationship with EIWB [45]. Hu et al. found that EIWB is mainly driven by economic and technological effects, with economic effects being more dominant [46]. In addition, other studies have explored the extent of the role of industrial structure, urbanization, technological progress, and foreign investment on EWP through LMDI, regression models, and spatial econometric models [41,43,47,48,49], but with different conclusions. 

### 2.3. Research Gaps

In summary, the existing studies of EWP still need further improvement. In terms of research scales, they mainly focus on national, regional, and urban scales, and research on small and medium scales such as the county level still needs to be strengthened. Insufficient attention is given to large river basins and artificial reservoir areas. In addition, there are relatively few EWP studies based on a strong sustainability research paradigm. Regarding research methods, most existing studies treat the whole production process as a “black box” for single-stage EWP measurement and cannot identify the validity of phasing. In addition, studies that use radial and non-radial epsilon-based measure (EBM) models are also rare. In the construction of the indicator system, only negative ecological inputs such as environmental pollution and resource consumption are usually considered, ignoring positive inputs such as capital [27,42]. At the same time, existing studies using DEA methods fail to address the problem of the small number of DMUs and too many input–output indicators, resulting in less accurate measurement results [50,51]. Given this lack of accuracy, this paper incorporates capital inputs into the EWP evaluation index system and uses PCA-DEA and network super-efficiency EBM models to dynamically measure the EWP of 26 counties (districts) of the TGRA from 2010 to 2020 and analyze their spatial and temporal patterns and influencing factors. 

## 3. Materials and Methods

### 3.1. Materials

#### 3.1.1. Study Area

**Figure 1:**
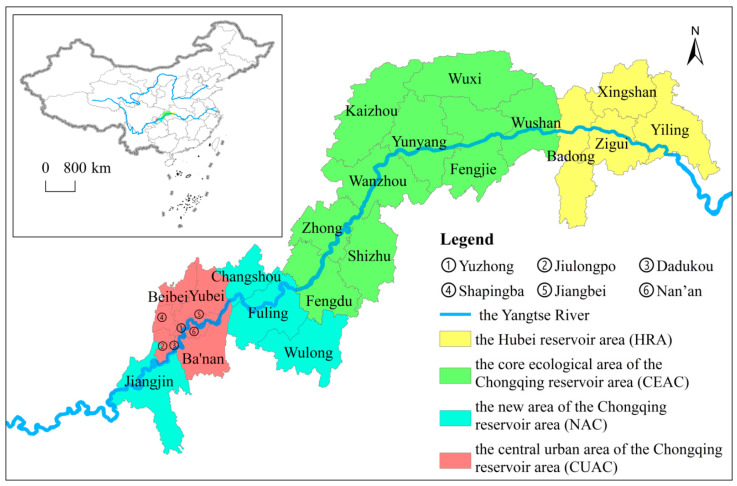
Study area.

The TGRA is the area that was inundated and has the task of migration due to the construction of the Three Gorges Dam Project (the world’s largest artificial water-conservancy project) [16], with a total area of 59,000 km^2^, including 26 counties (districts) under the jurisdiction of Chongqing City, Enshi Tujia and Miao Autonomous Prefecture, and Yichang City (Figure 1). The TGRA is located in the transition zone where China’s east–west terrain and north–south climate meet, with a forest coverage rate of more than 50%, and is an important ecological barrier area and a key zone for biodiversity conservation in the Yangtze River economic belt [15,16]. Due to the impact of the Three Gorges Dam Project construction, geological disasters such as mudslides, landslides, and earthquakes occur frequently; the ecological environment is fragile and difficult. Exploring how the TGRA can achieve optimal economic and well-being outputs under certain ecological constraints will provide an example of sustainable development in large river basins and artificial reservoir areas.

It divides the 26 counties (districts) of the TGRA into four major regions based on the current status of administrative divisions and a master plan for the construction of the Chengdu-Chongqing economic circle in Southwest China. They are the Hubei reservoir area (HRA) (Yiling District, Xingshan County, Zigui County, and Badong County), the core ecological area of the Chongqing reservoir area (CEAC) (Wushan County, Wuxi County, Fengjie County, Yunyang County, Kaizhou District, Wanzhou District, Zhong County, Fengdu County and Shizhu County), the new area of the Chongqing reservoir area (NAC) (Fuling District, Changshou District, Jiangjin District, and Wulong District), and the central urban area of the Chongqing reservoir area (CUAC) (Yuzhong District, Beibei District, Shapingba District, NanAn District, Jiulongpo District, Dadukou District, Jiangbei District, Yubei District, BaNan District). It should be noted that in 2016, Wulong County and Kai County in Chongqing were renamed Wulong District and Kaizhou District, respectively. Although the names were changed, the administrative area was not adjusted. To ensure the consistency of the study, the unified use of the adjusted administrative division name.

#### 3.1.2. Framework

EWP is decomposed into two stages of realization to open the black box of the transformation process of ecological inputs and well-being outputs [42,44,52,53]. In the first stage, capital inputs are used as intermediate means to transform into undesired (environmental pollution) and desired (economic outputs) outputs through ecological inputs (resource consumption) [27,42]. That is, eco-economic efficiency; in the second stage, the intermediate variables (economic outputs) are used as inputs transformed into integrated well-being outputs, that is, economic well-being efficiency. The formula is as follows:

(1) $$EWP=\frac{HDI}{EF}=\frac{GDP}{EF}*\frac{HDI}{GDP}\to EWP=\frac{WBs}{EI}=\frac{GDP}{EI}*\frac{WBs}{GDP}$$

**Figure 2:**
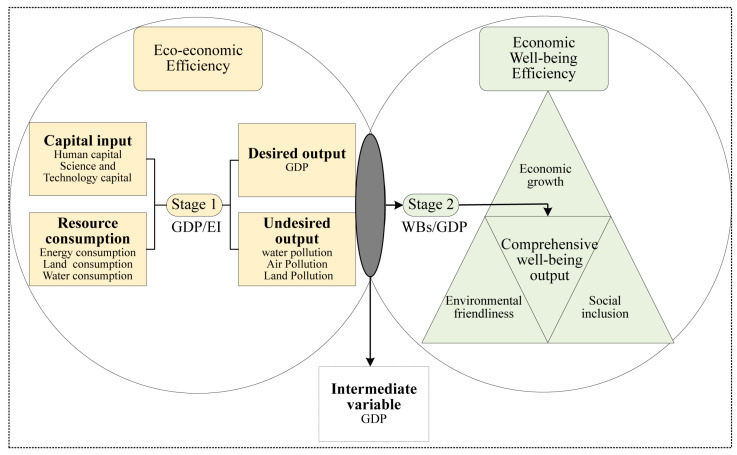
Eco-economic efficiency and economic well-being efficiency transformation system of EWP in TGRA.

In Equation (1): *EWP* represents ecological well-being performance; *HDI* (human development index) represents the objective level of well-being; *EF* (ecological footprint) represents the natural consumption level; *EI* (ecological input) is ecological input per capita; *GDP* (gross domestic product) represents economic growth; and *WBs* (well-beings) represents the comprehensive well-being level, including economic growth, social inclusion, and environmental friendliness. Based on Equation (1), this paper proposes a research framework for a two-stage EWP transformation system (Figure 2). It constructs an EWP evaluation index system according to the two stages.

#### 3.1.3. Evaluation Index System

EWP is an inheritance and innovation based on eco-efficiency [44], so the evaluation index system in this paper is based on the strong sustainability concept, and is constructed by combining two stages under the principles of scientific, systematic, operability, and typicality, drawing on the evaluation index systems of existing eco-efficiency and EWP literature (Table 1).

Regarding ecological inputs, the current academic community mostly uses negative indicators such as environmental and resource categories to characterize ecological inputs [27,42]. In this paper, we argue that ecological inputs should also introduce some capital-based positive indicators, which are helpful to comprehensively reflect regional EWP and beneficial for decision-making institutions to formulate corresponding policies. Therefore, based on Xiao et al. and Wang et al., human capital and science and technology capital indicators are introduced, which are characterized by per-capita education expenditure and per-capita science and technology expenditure, respectively [27,42]. In the ecological resource consumption section, the resource constraints of the TGRA were comprehensively considered, and the per-capita built-up area was selected to characterize land consumption, per-capita industrial energy consumption above the scale to characterize energy consumption, and per-capita water consumption to characterize water consumption. In the first stage, environmental pollution during the transformation process will affect the comprehensive well-being level [42]. Therefore, this paper uses environmental-pollution indicators as undesired outputs, and industrial wastewater, industrial waste gas, carbon emissions per capita, and industrial solid waste are chosen to characterize them. The accuracy of these indicators is confirmed by the studies of Xiao et al. and Bian et al. [27,43].

The first stage is the conversion of ecological inputs into economic outputs, so the expected output is the economic benefit indicator, GDP per capita was selected as the expected output indicator to measure economic benefits, and it is also the intermediate variable, i.e., the economic input in the second stage [42,52].

The second stage is the comprehensive well-being output indicator, mostly selected from the HDI in existing studies, but the HDI ignores the importance of environmental well-being [27]. Environmental well-being is an added goal proposed by the SDGs. Therefore, this paper combines the HDI and SDGs and uses 11 indicators in three dimensions, namely, economic growth, social inclusion, and environmental friendliness, to measure the comprehensive well-being outputs. Among them, disposable income per capita is often considered a reasonable indicator to characterize economic growth; thus, the disposable income per capita of urban and rural residents is chosen to represent. Social inclusion refers to the relevant indicators of HDI; from the level of education, health, and social security, the average years of education, the number of health technicians per 1000 population, per-capita health expenditure, per-capita social security and employment expenditure are chosen to represent. Environmental friendliness, in contrast, refers to SDGs-related indicators from the perspective of environmental protection, level, and response; the per-capita expenditure on energy conservation and environmental protection, forest coverage rate, per-capita park green area, the percentage of good days, and urban domestic sewage treatment rate are selected.

#### 3.1.4. Data Source and Processing

Since the Three Gorges Dam Project migration ended around 2010, this paper defines the study period as 2010–2020 based on data operability and consistency principles. The data in the evaluation index system are primarily drawn from the Statistical Yearbooks (2011–2021) and Statistical Communiqué on the National Economic and Social Development of the 26 counties (districts) (2011–2021) in the study area, as well as the *Hubei Provincial Statistical Yearbook* (2011–2021), *Chongqing City Statistical Yearbook* (2011–2021), *Yichang City Statistical Yearbook* (2011–2021), and *Enshi Tujia and Miao Autonomous Prefecture Statistical Yearbook* (2011–2021). The China Carbon Accounting Database https://www.ceads.net.cn/ (accessed on 10 November 2022), the water resources bulletins of each district and county, and the China County Construction Statistical Yearbook were also used to acquire data on carbon emissions, water usage, and built-up area. Each indicator is described by per capita or percentage, and the per-capita value is converted depending on the resident population to remove the impact of population size on the findings. Years of education per capita (AEY) is described the treatment of indicators by referencing the calculation technique in the Human Development Report of the United Nations Development Programme (UNDP) [43]: AEY = (6 × $P_{1}$ + 9 × $P_{2}$ + 12 × $P_{3}$ + 16 × $P_{4}$)/($P_{1}$ + $P_{2}$ + $P_{3}$ + $P_{4}$), where $P_{1}$, $P_{2}$, $P_{3}$, and $P_{4}$ represent the number of students enrolled in elementary, junior high school, senior high school, and university. Additionally, the year-to-year missingness of individual indicators was obtained by interpolation and extrapolation estimation with Stata MP 16.0 (Beijing UoneInfo&Tech Co., Ltd., Beijing, China).

### 3.2. Methods 

#### 3.2.1. PCA-DEA Analysis

The current measurement of EWP mainly includes SFA and DEA [57]. Compared with SFA, DEA has the advantages of no prior determination of functional relationships, non-subjective weighting, and no requirements on the magnitude of input–output indicators [52], which makes the measurement results more objective and more widely used. However, the DEA method has strict requirements on the number of input–output indicators and DMUs (i.e., the number of DMUs is not less than three times the sum of the number of indicators or greater than the product between the number of input and output indicators [50,51]). If the number of DMUs is too small and the number of input and output indicators is too large, the accuracy of the measurement results is easily reduced. However, in practical application, if there are too few evaluation indicators, it will not comprehensively reflect the EWP level. Principal component analysis (PCA) can better solve this difficulty by reducing the dimensionality of multiple original indicators and replacing them with extracted mutually independent composite indicators (principal components), each of which can be expressed as a linear combination of the original indicators and linearly independent between each principal component, thus achieving a reduction in the number of indicators and ensuring the accuracy of DEA measurement. Therefore, this paper uses a combination of PCA and DEA to measure EWP.

#### 3.2.2. Network Super-EBM Model and DEA Window Analysis

Data envelopment analysis (DEA) was proposed by Charns and Cooper (1978) [58]. Traditional DEA models (BCC model [59], CCR model [58], and Slack-based Measure (SBM) model [60]) suffer from the inability to cover slack variables or to handle variables with both radial and non-radial characteristics [61], which can easily lead to imprecise measurement results. Therefore, Tone et al. further proposed the Epsilon-based Measure (EBM) model that integrates radial and non-radial characteristics to improve the accuracy of DEA model measurement [62]. However, these models treat the whole production process as a “black box” and cannot identify the phase-by-phase validity. For this reason, Tavana et al. proposed a network EBM model that allows dividing the entire production process into multiple stages, enabling the evaluation of the overall DMU efficiency while evaluating the efficiency of each stage inside its “black box” [63].

The network EBM model helps open the county’s “black box” of the ecological well-being transformation process. In this study, the network EBM and super-efficiency model are chosen to solve the problems of undesired output indicators and multiple effective DMUs that cannot be ranked in the evaluation index system [64]. Based on Long [65], this paper uses the Network Super-Efficiency EBM (Network SEBM) model that considers undesired outputs under the assumption of variable payoffs of scale for the EWP measurement of TGRA, and the expression is as follows:

(2) $$\begin{array}{l} \min\rho_{se}=\frac{\sum_{h=1}^{H}W_{h}\left(\theta^{h}-\varepsilon_{x}^{h}\sum_{i=1}^{m_{h}}\frac{w_{i}^{h-}s_{i}^{h-}}{x_{i_{0}}^{h}}\right)}{\sum_{h=1}^{H}W_{h}\left(\varphi^{h}+\varepsilon_{y}^{h}\sum_{r=1}^{s_{h}}\frac{w_{r}^{h+}s_{r}^{h+}}{y_{r_{0}}^{h}}\right)}\\ s.t.\left\{\begin{array}{l} \sum_{j=1,j\neq k}^{n}x_{ij}^{h}\lambda_{j}^{h}+s_{r}^{h-}=\theta^{h}x_{i_{0}}^{h},i=1,\ldots ,m_{h},h=1,\ldots ,H\\ \sum_{j=1,j\neq k}^{n}y_{rj}^{h}\lambda_{j}^{h}-s_{r}^{h+}=\varphi^{h}y_{r_{0}}^{h},r=1,\ldots ,s_{h},h=1,\ldots ,H\\ \sum_{j=1,j\neq k}^{n}z_{pj}^{\left(k,h\right)}\lambda_{j}^{k}=\sum_{j=1,j\neq k}^{n}z_{pj}^{\left(h,k\right)}\lambda_{j}^{h},p=1,\ldots ,l_{h},k=1,\ldots ,H\\ \lambda_{j}^{h}\geq 0,s^{h-}\geq 0,s^{h+}\geq 0,j=1,\ldots ,n,j\neq k \end{array}\right. \end{array}$$

In Equation (2): $w_{i}^{h}$ denotes the weight of the *i*th input of the *h*th node and satisfies $\sum_{i=1}^{m_{h}}w_{i}^{h}=1$; $s^{h}$ denotes the slack variable of the *i*th input of the *h*th node; $\theta^{h}$, $\varphi^{h}$ and $\varepsilon_{x}^{h}$ denote the planning parameters of the radial part; and $w_{h}$ denotes the importance of the *h*th node determined by the decision maker. In this paper, the first and second stages are considered equally important, so each node has the same weight. The overall efficiency is considered DEA valid only if the efficiency of each stage is valid [65]. 

Since the Network SEBM model can only measure the efficiency of each DMU on a period, the efficiency of DMUs in different periods is not comparable. The DEA window analysis method can increase the number of evaluated DMUs and compare the efficiency of DMUs in both horizontal and vertical dimensions, thus reflecting the dynamic changes in efficiency [42]. Therefore, in this paper, the window DEA analysis method is used to measure the EWP of TGRA and to analyze the differences in EWP and the trend of efficiency changes among counties (districts). The window width also needs to be set before the window DEA analysis, and based on Wang et al. and Cullinane et al., we set the window width to 3 in our study [42,66].

#### 3.2.3. Geodetector

Geodetector is a new statistical method developed by Professor Wang to detect spatially stratified heterogeneity and reveal the driving forces behind it [67,68]. Geodetector can be obtained from this URL (http://geodetector.cn/) (accessed on 13 November 2022). It consists of four-factor detection modules: factor detector, interaction detector, risk detector, and ecological detector. The method has been widely used in the literature [16,69,70,71]. In this paper, the factor detector and interaction detector in the Geodetector model are used to identify the main influencing factors and their interactions that affect the spatial variation in EWP in the TGRA.

(1) Factor detection mainly analyzes the extent to which different influencing factors explain the spatial pattern of EWP in the study area [71], which is calculated as follows:

(3) $$q=1-\frac{1}{N\sigma^{2}}\sum_{h=1}^{L}N_{h}\sigma_{h}^{2}$$

In Equation (3): q is the degree of explanation of the influence factor on EWP; L denotes the number of regions; $N_{h}$ and N are the number of cells in layer h and the whole study area, respectively; $\sigma_{h}^{2}$ and $\sigma^{2}$ denote the variance of the sample in layer h(h=1,2,3,…,L) and the whole area, respectively. q takes values in the range of [0, 1], and the closer the value is to 1, the greater the influence of the factor on EWP, and vice versa is weaker [67].

(2) Interaction detection analysis of whether the two different factors acting together enhance or diminish the effect on the spatial pattern of EWP in TGRA [67]. The classification of the relationship between the two factors is shown in Table 2.

## 4. Research Process and Results

### 4.1. Research Process

Using the PCA-DEA method, the Network SEBM model was selected to measure and compare the EWP of 26 counties (districts) within the TGRA. Since there are 21 indicators in the evaluation index system and only 26 decision units, the requirement that the number of decision units is not less than three times the sum of input–output index data cannot be satisfied [50]. To ensure the accuracy of DEA measurement, it is necessary first to reduce the dimensionality of multiple input and output indicators by the PCA method, and finally obtain a comprehensive capital input indicator, a comprehensive resource consumption indicator, a comprehensive environmental pollution indicator, and a comprehensive well-being output indicator. Before conducting the PCA again, the four types of indicators need to be tested for applicability, mainly through KMO and Bartlett’s test (Table 3) (it is generally believed that only when the KMO value is greater than 0.5 and the probability value is less than 0.05 is it suitable for principal component analysis [72]). The PCA was completed using SPSS Statistics 22 (IBM, Armonk, NY, USA). The correlation between the two indicators of capital input is not high (KMO value of 0.484) and does not meet the requirements. Therefore, the two indicators of capital input are not suitable for PCA and can be standardized and directly substituted into the DEA model, while the KMO values of resource consumption, environmental pollution, and comprehensive well-being output factor analysis are 0.531, 0.604, and 0.694, respectively. The *p*-value of Bartlett’s sphericity test probability is less than 0.001, indicating that the three are suitable for PCA. There are 1/1/3 principal components for resource consumption, environmental pollution, and comprehensive well-being output indicators, which replace 3 resource consumption indicators, 4 environmental-pollution indicators, and 11 comprehensive well-being output indicators, respectively, plus two capital input indicators for a total of 5 new indicators.

In this paper, after using PAC to determine the new input–output indicators, the network SEBM model and window DEA analysis was then applied to measure the overall and two-stage efficiency of the 26 counties (districts) of the TGRA from 2010 to 2020 with the help of Maxdea Ultra 9 software (http://www.maxdea.cn/maxdea_cn.htm) (accessed on 15 November 2022). According to a related study [25], EWP was divided into five stages: excellent performance (EWP ≥ 1), high performance (1 > EWP > 0.8), relatively high performance (0.8 > EWP > 0.6), relatively low performance (0.6 > EWP > 0.4) and low performance (0.4 > EWP > 0).

### 4.2. The Overall Efficiency Status of EWP in the TGRA

#### 4.2.1. Temporal Evolutionary Characteristics of the Overall Efficiency of EWP in the TGRA

**Figure 3:**
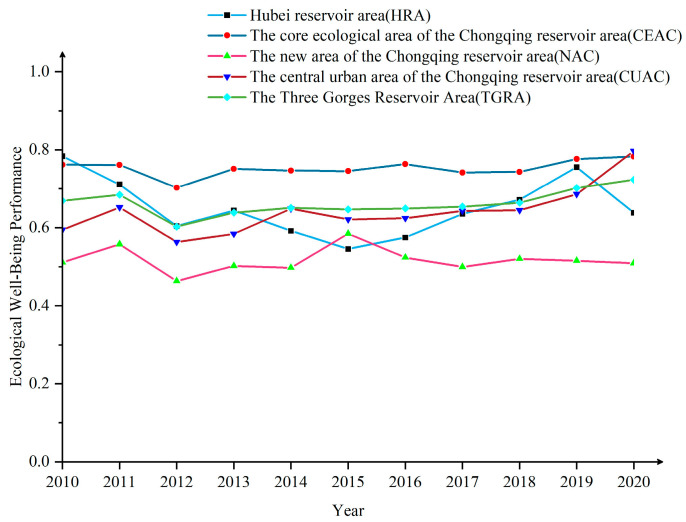
The trend of EWP in the TGRA, 2010–2020.

From 2010 to 2020, the EWP of the TGRA showed a “V”-shaped fluctuation trend (Figure 3), from 0.669 to 0.723, with an average annual growth rate of 0.79%, indicating that the efficiency of the TGRA’s ecological inputs to well-being outputs gradually improved during 2010–2020. Among them, the lowest value of EWP appeared in 2012, which may be caused by the convening of the 18th National Congress of the Communist Party of China, which established the strategy of ecological civilization and, to a certain extent, influenced the development model of high pollution and high energy consumption, causing a brief decline in local EWP. With the upgrading of industrial structures, the enhancement of environmental regulations, and the deepening of the concept of strong sustainability, the local EWP has gradually improved. Nevertheless, the current EWP of the TGRA is only 0.723, which is an ineffective state, and the conversion of ecological inputs into well-being outputs is less efficient and needs to be further improved in general.

**Figure 4:**
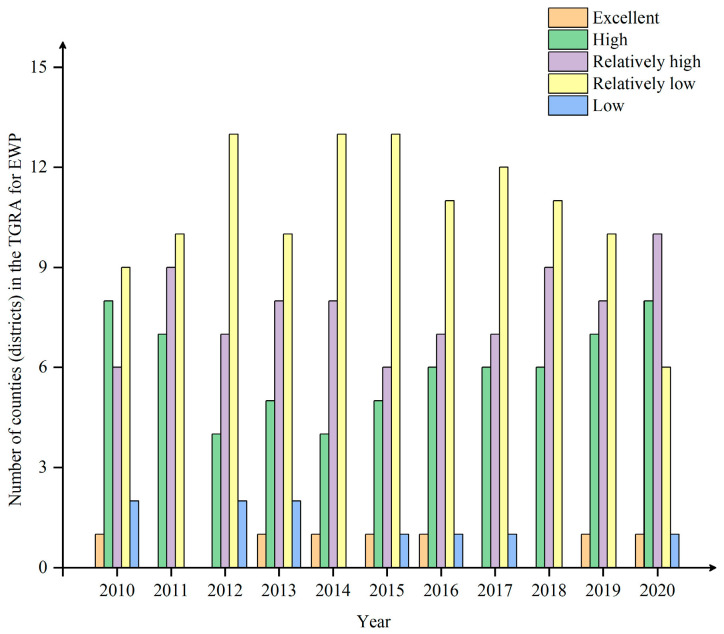
Number of EWPs in the TGRA by grade, 2010–2020.

For the four major regions, CEAC > HRA > CCC > NAC, with mean values of 0.752, 0.651, 0.642, and 0.517, respectively. Among them, CEAC is significantly higher than other regions, consistent with its excellent ecological environment, low energy consumption, and low pollution. The long-term position of NAC at the bottom of the list, showing an inverted “V” structure, may be related to the fact that the NAC took over a large number of relocated industries in the early period, which led to a rapid increase in GDP and boosted the local EWP. Then, as energy consumption and pollution emissions intensified and economic growth slowed down, the local EWP experienced a significant decline. Unlike the three regions in Chongqing, the HRA shows a rapid decline and a rapid rise, probably due to the low level of economic development in the HRA, which was still in the early stage of industrialization before 2015. The rapid development of industry brought high energy consumption and pollution, which led to the decline in EWP. As industrialization entered the middle and late stages, industrial energy consumption and pollution decreased, per-capita GDP increased significantly, the ecological environment improved, and the EWP gradually rose. Finally, from the number of different levels of EWP (Figure 4), the 26 counties (districts) of the TGRA show an olive shape, i.e., there are few excellent and low-performance counties (districts). The number of relatively low, relatively high, and high counties (districts) accounts for approximately 94% of the total, which again indicates that the overall level of EWP in the TGRA is low and still needs further improvement.

**Figure 5:**
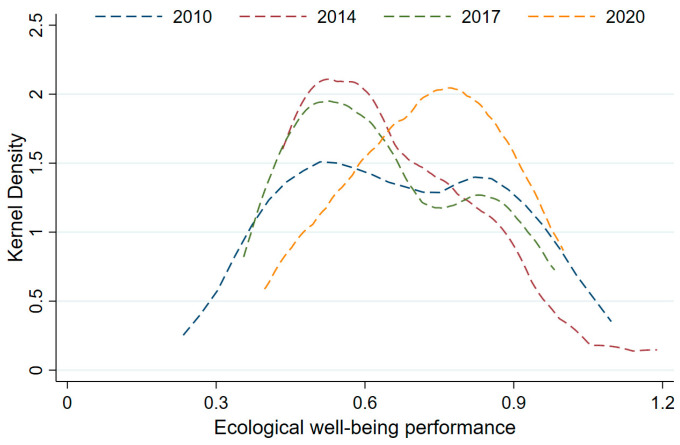
The kernel density of EWP in the TGRA, 2010–2020.

For comparison, the EWP of the TGRA in 2010, 2014, 2017, and 2020 was selected to plot the kernel density function in this study (Figure 5). The kernel curve moves to the right with the main peak and shows an overall upward trend, while the waveform shows a transition from a double peak to a single peak, indicating that the EWP of the TGRA is gradually improving and the polarization has weakened. In addition, the distribution curve indented from the right to the left side, and the double-tailed extension continued to become narrower, indicating that the internal variation in the TGRA was slowing down.

#### 4.2.2. Spatial Evolution Characteristics of the Overall Efficiency of EWP in the TGRA

**Figure 6:**
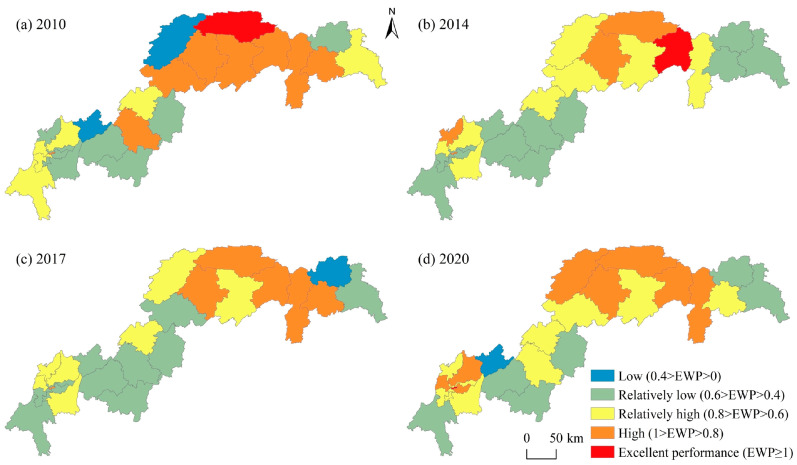
Spatial Distribution of EWP in the TGRA.

Regarding spatial distribution characteristics (Figure 6), the EWP in the study area generally showed a distribution pattern of high in the east and low in the west, with higher performance values in CEAC and HRA and lower performance values in CUAC and NAC. The top five regions in terms of mean value are mainly Wushan County (1.013), Yuzhong District (0.957), Wuxi County (0.923), Yunyang County (0.906), and Badong County (0.857), of which four counties (districts) are located in the eastern region. The lowest mean values are in Changshou District and Xingshan County, with values of 0.446 and 0.439, respectively, in low-efficiency areas. The reason may be that Wushan County, Wuxi County, Yunyang County, and Badong County have relatively backward industrial conditions, less energy consumption, and environmental pollution, as well as better ecological resource endowment and high efficiency of EWP. Yuzhong District, on the other hand, is located in the central urban area of the Chongqing reservoir area, with a developed economy, more advanced technology, excellent public infrastructure, and social well-being protection system, which to a certain extent improves the efficiency of the local eco-economic stage and economic well-being stage transformation. It is worth noting that the EWP levels of Fuling District, Changshou District, Yubei District, NanAn District, Jiulongpo District, Dadukou District, and Jiangbei District, which have better economic development, are all less than 0.56 and have not increased significantly, which are inefficient areas, indicating that the increase in ecological inputs brought by economic development has not led to a subsequent significant increase in well-being levels and has failed to decouple economic growth from ecological consumption. Therefore, it also shows to a certain extent that the level of EWP not only depends on the level of economic growth but also has a greater relationship with ecological resource consumption and environmental pollution.

### 4.3. The Substage Efficiency Status of the EWP in the TGRA

#### 4.3.1. Temporal Evolution Characteristics of the Substage Efficiency of EWP in the TGRA

**Figure 7:**
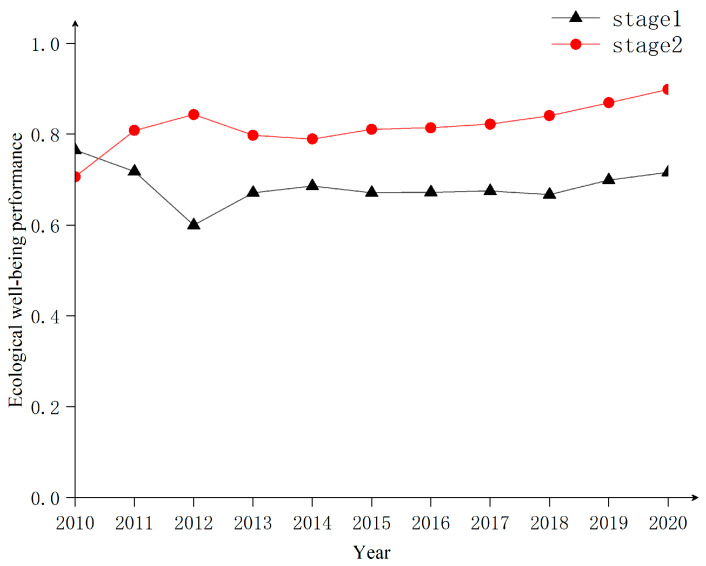
Two-stage EWP trends in the TGRA, 2010–2020.

Both eco-economic and economic well-being efficiency show an increasing trend from 2010 to 2020 (Figure 7), indicating that ecological inputs and well-being outputs in the TGRA are gradually improving. However, both stages are less than 1 in 2010–2020, which is in the DEA invalid state, indicating that the relationship between economic development and environmental protection is not coordinated, and the level of economic development and well-being enhancement is not high. Among them, the efficiency value of the first stage is lower than that of the second stage for an extended period (the mean values are 0.686 and 0.818, respectively); the growth rate of the first stage is also lower than that of the second stage and the gap between them is gradually increasing. This result indicates that the overall economic output of the TGRA is insufficient, and the eco-economic efficiency is the main reason that restricts its EWP improvement, which is consistent with the conclusion reached by Wang et al. in the study of Poyang Lake [42]. The lack of eco-economic efficiency also indicates that ecological resource consumption and economic growth have failed to achieve decoupled development. The current economic growth is still at the cost of inefficient ecological inputs. The reasons for this outcome may be caused by the low transformation efficiency of ecological resources in the TGRA as a whole and the insufficient support role of human and scientific resources in the transformation process. At the same time, the slowdown of economic growth in middle and late industrialization may also play a limiting role.

#### 4.3.2. Spatial Evolution Characteristics of the Substage Efficiency of EWP in the TGRA

Regionally (Table 4), the eco-economic efficiency of all four regions is lower than the economic well-being efficiency, but there are differences among regions and their improvement strategies are distinct. NAC is in first place in terms of economic well-being efficiency. However, the eco-economic efficiency is in last place, probably because the high pollution and high emissions of NAC affect the conversion efficiency of the eco-economy, thus limiting the improvement in EWP, so it is urgent to control the environment and upgrade the industrial structure in this area. The CUAC, on the other hand, is at the bottom of the list of economic well-being conversion efficiency due to its high population density, which puts additional pressure on the regional environment and public social infrastructure. Therefore, the CUAC should further improve the level of comprehensive regional well-being while improving the efficiency of the ecological economy. The CEAC and HRA have a good ecological environment and high economic well-being efficiency. However, the economic development level is low in the TGRA, which affects the overall EWP improvement and needs to strengthen economic construction and improve eco-economic efficiency further.

Of the 26 counties (districts), only Wushan County and Yuzhong District have a mean value of well-being performance at stage 1 greater than 1, achieving a relatively effective DEA, indicating that these two regions have good results in converting ecological inputs into the economy. However, the efficiency of converting the economy into well-being is still insufficient and needs to be further improved. The remaining regions have a mean value of less than one at two stages, an ineffective DEA state. In some regions that did not achieve DEA relatively effectively, there is a trend of efficiency reduction, and overall, most of the regions have a low level of EWP. The ecological well-being situation in the TGRA area is still not optimistic. 

### 4.4. Analysis of the Improvement in Input–Output Indicators of EWP in the TGRA

To further clarify the actual situation of excessive regional inputs or insufficient outputs and to provide policy recommendations for each region, the input–output indicators of the EWP of 26 counties (districts) in the TGRA were analyzed in 2020 as an example (Table 5). A total of 25 counties (districts) in DEA ineffective have different degrees of redundancy in capital input, resource consumption, and environmental pollution. In contrast, some regions have insufficient GDP per capita and well-being output. Combined with the specific results measured in Table 5, this paper analyzes and proposes improvement paths through each region as follows:

1. Although CEAC ranks high in EWP, the growth rate slows, and the insufficient GDP output per capita is an important reason. Therefore, ecological tourism and green industries should be vigorously developed to improve the economy by relying on the advantages of ecological resources.
2. There is more room for improvement in resource consumption and environmental pollution in NAC. Optimizing energy structures, developing green and clean energy, accelerating industrial structure upgrading, and enhancing environmental protection are all directions for its improvement.
3. The EWP of the CUAC is at the forefront. However, the high population density of the region also imposes a burden on public infrastructure and the relative lack of comprehensive well-being outputs per capita. Therefore, on the one hand, regional planning and management should be strengthened to transform the population advantage into economic development; on the other hand, the construction of public infrastructure should be enhanced to improve well-being.
4. The HRA, on the other hand, has put greater pressure on regional finance due to excessive investment in human capital and science and technology capital, which has affected the further enhancement of EWP. Human and science and technology investment should be appropriately reduced in the future to avoid creating a large amount of debt [31]. In addition, there are problems such as high environmental pollution and insufficient GDP output per capita, which also need to strengthen the supervision of the environment further and develop green ecological industries according to local conditions.

### 4.5. The Influencing Factors in the TGRA

#### 4.5.1. Dominant Factor Identification

In this paper, EWP was used as the explanatory variable, and the explanatory variables were selected from the economic development level, policy system, social-life condition, and natural environment to establish the index system (Table 6). The factors influencing the spatial differentiation pattern of EWP in the study area were detected and interacted with each other using the Geodetector. 

The explanatory variables were introduced into the Geodetector model to detect the influence of the spatial pattern of EWP in the TGRA in 2010, 2014, 2017, and 2020. The results showed (Table 7) that the level of economic development level > social-living conditions > policy system > natural environment, indicating that the level of economic development plays a dominant role in the pattern of EWP in the TGRA, and the social-living conditions and policy system play a significant role in the spatial pattern of EWP in the TGRA. The spatial pattern of EWP in the study area plays an important role, and the natural environment has a relatively weak influence.

In general, the natural environment’s influence on EWP’s spatial pattern tends to weaken, while the influence of socioeconomic factors gradually increases. The influence of X1, X2, and X3 are on the rise, while X1 has been in first place for a long time, indicating that the level of economic development level is the dominant factor in the spatial pattern of EWP in the TGRA. X4 shows a trend of rising and then falling, indicating that the early level of science and technology will promote the conversion efficiency of the eco-economic stage. However, with the reality of tightening resources, the influence of the level of science and technology is gradually weakening. The influence of X5 and X6 also shows a significant increase, indicating that the influence of the policy system and the social-living condition on the spatial pattern of EWP in the TGRA is gradually increasing, and the comprehensive well-being level of the region will be effectively improved by increasing the investment in livelihood areas such as health, social security and social well-being undertakings, education and natural environment protection in the TGRA, and driving the construction of urban infrastructure. The influence of X8 and X9 on EWP gradually decreases over time, and their role in enhancing EWP is smaller due to the better natural environment base in the TGRA and the smaller differences between counties (districts). Notably, the influence of X7 on the spatial variation in EWP is significantly higher. Related studies indicate that moderate regional population concentration is beneficial to the efficiency of resource utilization [73]. However, it is also necessary to prevent the urban disease brought on by the excessive increase in population density, which in turn affects the level of EWP.

#### 4.5.2. Interaction Detector Results

**Figure 8:**
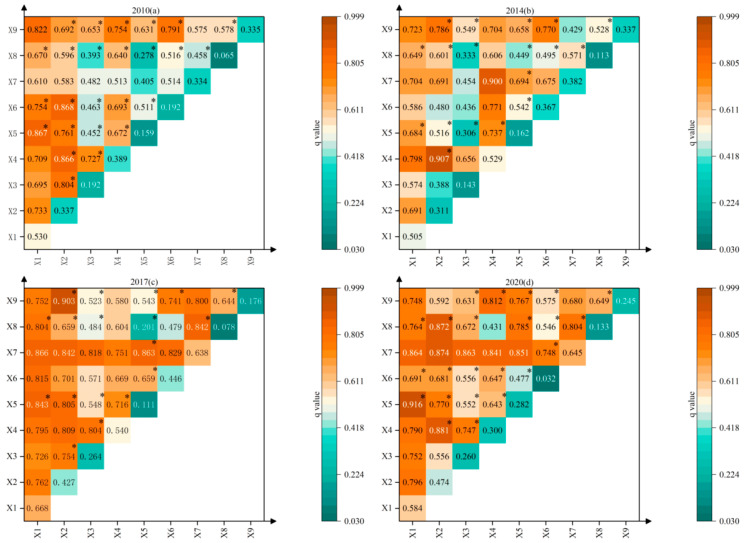
Interaction detector results of EWP in the TGRA in 2010, 2014, 2017, and 2020. Note: * is non-linear enhancement, the rest is two-factor enhancement.

The nine types of influencing factors were subjected to interaction detector analysis (Figure 8). The results showed that the interaction between any two factors showed a two-factor enhancement or nonlinear enhancement. There was no mutually independent or weakening relationship; that is, the interaction between any two factors had a greater impact on EWP than a single factor. The spatial evolution of EWP in the TGRA was affected by the joint action of multiple factors, and the higher the q value of the interaction, the greater the degree of influence of the interaction between its corresponding two factors on EWP.

From 2010 to 2020, the q-values of the interaction between X1 and all other factors are higher (q > 0.505), which is related to the higher single-factor explanatory power of GDP per capita on the spatial distribution of EWP and indicates that the enhancement of the economic development level will effectively improve the level of EWP. Second, the interaction between X2 and its residual factors indicates that the development of the EWP spatial pattern in the TGRA is mainly influenced by GDP per capita, industrial structure, and other factors. In addition, the influence of X8 and X9 is relatively weak in the single-factor detection (q mean value is 0.195 and 0.27), while the influence of X8 increases nonlinearly after interacting with X1, X2, and X7, and the q value reaches above 0.43 after interaction; the influence of X9 after interacting with X3, X5, X6, and X8 also showed a significant increase in influence, with q values above 0.52 after the interaction. It shows that the influence of the natural environment on the spatial pattern of EWP can only be fully reflected under joint action with the policy system and social-living conditions. It also reflects that the level of EWP cannot be effectively improved only by improving X8 and X9. In addition, the influence of X3 and X5 is at the bottom of the single-factor detection, mainly showing indirect influence, but the q-value increases significantly after interacting with other factors, especially after combining with X2, X4, X6, X8, and X9. 

## 5. Discussion

### 5.1. Discussion of Findings

Based on a strong sustainability perspective, this paper measures the overall and two-stage EWP in the TGRA by PCA-DEA, the network SEBM model, and other methods, and analyzes its influencing factors by using Geodetector. Compared with previous studies, the contributions of this study are as follows:

First, in terms of research methods, Xia et al., Wang et al., and Hu et al. used network super-efficiency SBM model and two-stage network DEA model for BTH, Poyang Lake, and the Yangtze River Delta, respectively [44,73,76]. Compared with their studies, this paper combines PCA and DEA methods to solve the problem of imprecise results when the number of DMUs is small and the number of input–output indicators is too large [50,51]. Meanwhile, the network SEBM model is applied to evaluate EWP, which solves the problem that SBM cannot handle input and output variables with radial and non-radial characteristics at the same time [62]. Applying the PCA-DEA method and the network SEBM model can provide a methodological reference for future EWP research.

Second, regarding research cases, the TGRA selected for this study is somewhat typical because it is an artificial reservoir area formed by the construction of human super-engineering (the Three Gorges Dam Project). The construction of the Three Gorges Dam Project has changed the local natural environment and had many impacts on the local society and economy, forming a complex geographical system with social and ecological interactions. In addition, due to the large population, a small amount of land for construction, and a fragile ecological environment in the TGRA, there is a long-standing natural conflict between economic development and ecological protection, resulting in a more intense human-land conflict within this area. Although previous studies have focused on EWP in urban or key ecological areas [42,54], they have not paid sufficient attention to EWP in artificial reservoir areas under high human disturbance. This study has at least two implications for the empirical study of the TGRA: first, it is an important addition to the type of EWP study cases, and second, it has some implications for the EWP of other human super-engineering impact areas, especially for the construction of other large water facilities with strong insights.

Finally, this study also finds that the level of economic development plays a dominant role in the EWP pattern in the TGRA. In contrast, social-living conditions and policy systems play an important role in the evolution of the spatial pattern of EWP in the study area, and the natural environment has a relatively weak influence on it. Behjat and Tarazkar’s empirical study in Iran also showed that there is a positive relationship between economic development and IEWP, while energy consumption, demographic status, and environmental pollution are negative for improving IEWP [47]. This shows that the level of EWP not only depends on the level of economic development, but also has a strong relationship with ecological resource consumption, environmental pollution, and population growth. Interestingly, the relationship between economic development and EWP has also been specifically studied by related scholars. For example, He et al. found that there is a significant negative spillover effect of the level of economic development on EWP in Jiangsu Province [41]. Dietz et al. found that GDP per capita shows a U-shaped relationship with EIWB [45]. Zhu and Zhang found that the relationship between EWP and economic growth is an inverted U-shaped relationship, with the turning point occurring when GDP per capita reaches 3000 international dollars [77]. Thus, no uniform conclusion has been reached on the relationship between the two studies. For the TGRA, the degree of influence of its economic development level on EWP and the mechanism of its influence need to be further investigated in depth in future studies. In addition, it was found during the study that the Chinese government provided different amounts of policy and financial support to counties (districts) within the TGRA, which also played a key role in EWP’s evolution and spatial variation within the TGRA.

### 5.2. Shortcomings and Prospects

This study has achieved some useful conclusions in the study of EWP in the TGRA, but space for improvement remains.

1. In data acquisition, the evaluation indicators are all relatively single and relatively single panel data. In the future, multi-source heterogeneous data such as panels, remote sensing, and text can be used to enhance the comprehensiveness of evaluation indicators.
2. About the index system, because the academic community has not yet reached a consensus on the index system for evaluating the EWP of artificial reservoir areas, influenced by the accessibility of research data, the selection of evaluation indexes in this study may not be comprehensive enough, and there may be slight errors on the research results. In the future, several case sites of artificial reservoirs at different scales from international and domestic can be selected for comparative analysis to refine a universal evaluation index system and enhance the international applicability of the study. For example, Scandinavian countries, which are considered global leaders in sustainable development [78], could be strengthened in future research with comparative studies of these countries, to provide more insights for regions with lower levels of sustainable development.
3. The evolutionary process and driving mechanism of EWP of artificial reservoir areas can be analyzed from different scales, and the evolutionary rules and dominant factors of EWP of artificial reservoir areas at different scales can be explored to achieve mutual justification of research findings. In terms of influencing factors, the mechanisms of economic development level, government support policies, transfer funds, environmental regulations and industrial upgrading on EWP of artificial reservoir areas are not clear, and the studies on influencing factors are mostly conducted from a linear perspective. Future studies can consider using mediated effects models, spatial econometric models, and logarithmic mean divisia index (LMDI) to investigate the influence mechanisms of economic development level, government support policies, transfer funds, environmental regulations, and industrial upgrading on EWP in artificial reservoir areas, to better provide policy recommendations to decision-makers. We can also use panel Tobit models to examine the nonlinear characteristics to enrich related studies.

## 6. Conclusions and Policy Recommendations

### 6.1. Conclusions

The main conclusions of this research are as follows:

1. The overall level of EWP in the TGRA from 2010 to 2020 is low and varies widely among districts, failing to achieve effective DEA, but showing a favorable trend of year-on-year improvement. Spatially, the EWP in the TGRA shows a spatial distribution pattern of high in the east and low in the west, while the internal differences are gradually decreasing. The study also finds that the transfer of high pollution and high-energy-consuming industries from economically developed regions has brought adverse effects on the EWP of less economically developed regions.
2. By stages, eco-economic efficiency is significantly lower than economic well-being efficiency, and eco-economic efficiency is the main reason that restricts the improvement in EWP. Ecological resource consumption and economic growth in the TGRA have failed to achieve decoupled development, the current economic growth is still at the cost of inefficient ecological inputs, and the ecological well-being situation is not optimistic.
3. Based on the concept of strong sustainability, the study finds that there are two unsustainable situations in the TGRA, i.e., economically developed counties (districts) have high economic growth and well-being levels, but their EF exceeds the carrying capacity and fails to achieve decoupling of economic and social development from resources and environment; less economically developed counties (districts) have EFs that do not exceed the ecological carrying capacity but do not have economic growth and well-being levels that meet basic needs. The TGRA in general has the problem of excessive ecological input and insufficient per-capita GDP and well-being output, and targeted improvement measures should be formulated to promote sustainable regional development.
4. The level of economic development plays a dominant role in the EWP pattern of the TGRA, social-living conditions, and policy institutions play an important role in the evolution of the EWP spatial pattern of the study area, and the ecological environment has a relatively weak influence on it. However, the interaction of any two factors has a greater impact on the EWP than on individual factors. The level of EWP not only depends on the level of economic development but also has a greater relationship with the policy system, socio-economic conditions, and natural environment.

### 6.2. Policy Recommendations

Based on the findings of our study, some policy recommendations can be provided for local sustainable development.

First, a sustainable-governance model of cooperative governance should be constructed. Improving regional EWP and promoting regional sustainable development is a long-term systemic project that requires the combined efforts of government, enterprises, and society to realize a multifaceted cooperative governance model that is “government-led, enterprise supported and socially engaged”. Specifically, the government and the government should cross administrative barriers and actively seek cooperation to jointly improve regional EWP and avoid the transfer of high pollution and high-energy-consuming industries from economically developed regions, which will adversely affect the EWP of economically underdeveloped regions. Between the government and enterprises, the government should cooperate as an arranger and enterprises as a provider to improve various urban infrastructures and enhance the comprehensive well-being level. Meanwhile, the government provides financial and policy support for enterprises’ green transformation, and enterprises support the government’s environmental governance by paying taxes. Between enterprises and society, social organizations and consumers strengthen their concern and influence on environmental issues to promote the green transformation of enterprises, which can provide more well-being for society. Between government and society, the government can cooperate with social organizations to provide relevant social services. In addition, the European model of Corporatism in the Nordic countries generally has high overall sustainable development performance and balanced economic, social, and environmental performance [79]. This model, which emphasizes the balanced development of three types of organizations (government, business, and civil society), provides an example for many regions in the world from which local communities can learn relevant experiences and promote the construction of a sustainable-governance model of cooperative governance.

Secondly, improvement strategies are developed according to local conditions. As the four regions and 26 districts and counties in the TGRA have different levels of natural-resource endowment and economic development, there are different degrees of input redundancy and output deficiencies in capital input, resource consumption, environmental pollution, economic output, and comprehensive well-being output, so relevant measures should be taken to improve EWP according to local conditions. To address the redundancy of HRC capital inputs, local governments should appropriately reduce human and technological expenditures to avoid causing excessive local debt. In response to the excessive consumption of resources and serious environmental pollution in NAC, the state should formulate relevant laws and regulations to restrain the pollution behavior of enterprises; the local government needs to guide the upgrading of the industrial structure while strengthening environmental supervision; enterprises need to introduce energy saving and pollution control equipment to improve energy utilization efficiency; and community residents need to establish the awareness of resource conservation, environmental protection, and green travel. In response to the lack of GDP output per capita in the CEAC, the government should develop a green economy based on the advantages of ecological resources and reduce the blind investment in high-pollution and high-emission industrial enterprises. In response to the lack of comprehensive well-being output per capita in the CUAC, the national level should introduce relevant policies to consolidate the social well-being construction and protection system; the local government should reasonably allocate public service resources and strengthen the construction of public infrastructure such as medical care and education; and enterprises should enhance employee well-being and protect the reasonable rights of employees.

Thirdly, source management and post-pollution management are both important. Sustainable-development management requires not only end-of-pipe governance to control the deterioration of environmental conditions, but also changes in the development model at the source. For example, the national level needs to modernize the national governance system and governance capacity. Local governments should encourage the sharing economy to substantially increase the productivity of natural capital at the source of material production and consumption and to prevent and reduce the generation and disposal of waste afterward. The production and operation of industry, construction, transportation, and other enterprises should improve energy conversion efficiencies, e.g., using clean energy such as nuclear power, wind power, solar power, and natural gas to replace fossil energy, and prevent and reduce ex post CO_2_ emissions and disposal.

## Figures and Tables

**Table 1:** Evaluation index system.

Stage	Category	Level One Indicator	Level Two Indicator	Level Three Indicator	References
Stage 1 (Eco-economic efficiency)	Input indicators	Capital input	Human capital	Education expenditure per capita	[27,42]
			Science and Technology capital	Science and technology expenditure per capita	[27,42]
		Resource consumption	Energy consumption	Per-capita industrial energy consumption above the scale	[46,48,54]
			Land resource consumption	Built-up area per capita	[42,46]
			Water consumption	Water consumption per capita	[48,55]
	Undesired output	Environmental pollution	water pollution	Industrial wastewater emissions per capita	[27,56]
			Air pollution	Industrial waste gas emissions per capita, per-capita carbon emission	[46,56]
			Land pollution	Industrial solid waste generation per capita	[24,56]
Intermediate variable	Desired output	Economic benefits	GDP	GDP per capita	[42,52]
Stage 2 (Economic well-being efficiency)	Output indicators	Comprehensive well-being output	Economic growth	Per-capita disposable income of urban residents, per-capita disposable income of rural residents	[42,52]
			Social inclusion	Years of education per capita, health technicians per 1000 population, health expenditure per capita, social security, and employment expenditure per capita	[42,48,54]
			Environmental friendliness	Energy saving and environmental protection expenditure per capita, forest coverage rate, park green space per capita, excellent-air-quality rate, urban domestic sewage treatment rate	[41,48]

**Table 2:** Types of interaction between two covariates.

Graphical Representation	Description	Interaction
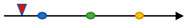	$q\left(X1\cap X2\right)<Min\left(q\left(X1\right),\;q\left(X2\right)\right)$	Nonlinear weakening
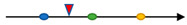	$Min\left(q\left(X1\right),\;q\left(X2\right)\right)<q\left(X1\cap X2\right)<Max\left(q\left(X1\right),\;q\left(X2\right)\right)$	Single-factor nonlinear weaking
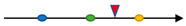	$q\left(X1\cap X2\right)>Max\left(q\left(X1\right),\;q\left(X2\right)\right)$	Two-factor enhancement
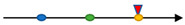	$q\left(X1\cap X2\right)=q\left(X1\right)+q\left(X2\right)$	Independent
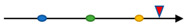	$q\left(X1\cap X2\right)>q\left(X1\right)+q\left(X2\right)$	Nonlinear enhancement

Note: $Min\left(q\left(X1\right),\;q\left(X2\right)\right)$: obtains the minimum value from $q\left(X1\right),\;q\left(X2\right)$; $Max\left(q\left(X1\right),\;q\left(X2\right)\right)$: obtains the maximum value from $q\left(X1\right),\;q\left(X2\right);q\left(X1\right)+q\left(X2\right):\;q\left(X1\right),\;q\left(X2\right)\;$ are summed; $q\left(X1\;\cap \;X2\right):\;q\left(X1\right),\;q\left(X2\right)$ interact.

**Table 3:** KMO, Bartlett’s test, and PCA.

Category	KMO	Bartlett Test of Sphericity			PCA			
		Approx. Chi-Square	df	*p* Value	Component	Total	%	Cumulative %
Capital input	0.484	73.096	3	0.000	-	-	-	-
Resource consumption	0.531	145.41	3	0.000	1	1.696	56.524	56.524
Environmental pollution	0.604	507.02	6	0.000	1	2.462	61.543	61.543
Comprehensive well-being outputs	0.694	2227.286	55	0.000	1	3.605	32.769	32.769
					2	2.927	26.608	59.378
					3	1.253	11.394	70.772

**Table 4:** Two-stage efficiency of EWP in the TGRA from 2010 to 2020.

DMU	2010		2014		2017		2020		Average	
	Stage 1	Stage 2	Stage 1	Stage 2	Stage 1	Stage 2	Stage 1	Stage 2	Stage 1	Stage 2
Kaizhou District	0.752	0.233	0.626	0.738	0.854	0.821	0.871	0.886	0.756	0.743
Wulong District	0.497	0.768	0.456	0.891	0.463	0.956	0.528	0.957	0.487	0.898
Fengdu County	1.000	0.679	0.588	0.802	0.488	0.819	0.626	0.880	0.660	0.814
Zhong County	1.000	0.528	0.798	0.786	0.843	0.790	0.814	0.879	0.803	0.817
Yunyang County	1.000	0.930	1.000	0.745	1.000	0.779	0.896	0.895	0.991	0.844
Fengjie County	1.000	0.689	0.916	0.674	0.758	0.813	0.696	0.872	0.853	0.749
Wushan County	1.000	0.948	1.228	1.026	1.000	0.911	0.966	0.951	1.037	0.962
Wuxi County	1.000	1.000	0.888	0.911	1.000	0.950	1.000	0.965	0.942	0.945
Fuling District	0.663	0.583	0.401	0.811	0.423	0.967	0.429	0.927	0.453	0.882
Wanzhou District	1.000	0.728	0.720	0.701	0.584	0.768	0.598	0.905	0.639	0.754
Shizhu County	0.447	0.657	0.465	0.799	0.435	0.921	0.499	1.000	0.467	0.839
Changshou District	0.335	0.837	0.497	0.870	0.367	0.945	0.318	1.000	0.387	0.913
Yubei District	1.000	0.591	1.000	0.621	1.000	0.640	0.889	0.758	0.930	0.659
BaNan District	0.522	0.645	0.632	0.800	0.673	0.766	0.734	0.861	0.615	0.784
Jiangjing District	0.595	0.767	0.511	0.746	0.536	0.776	0.518	0.840	0.585	0.810
Yuzhong District	1.000	0.797	1.000	0.898	1.000	0.967	1.000	1.000	1.000	0.921
Beibei District	0.527	0.716	0.913	0.829	0.597	0.809	0.714	0.903	0.683	0.828
Shapingba District	0.833	0.626	0.876	0.674	0.794	0.713	1.000	0.778	0.758	0.715
NanAn District	0.442	0.696	0.451	0.807	0.553	0.763	0.933	0.819	0.513	0.789
Jiulongpo District	0.749	0.624	0.521	0.689	0.567	0.697	0.853	0.791	0.588	0.721
Dadukou District	0.636	0.504	0.419	0.876	0.448	0.802	0.651	0.865	0.490	0.824
Jiangbei District	0.429	0.683	0.477	0.765	0.526	0.738	0.708	0.838	0.512	0.795
Xinshan County	0.508	0.777	0.422	0.777	0.305	0.863	0.379	0.989	0.399	0.814
Zigui County	1.000	0.805	0.554	0.734	0.903	0.815	0.739	0.887	0.775	0.801
Yiling District	1.000	0.642	0.566	0.821	0.433	0.757	0.384	0.930	0.582	0.810
Badong County	0.958	0.913	0.910	0.742	1.000	0.835	0.873	1.000	0.920	0.850
HRA	0.867	0.785	0.613	0.768	0.660	0.818	0.594	0.951	0.669	0.819
CEAC	0.911	0.710	0.803	0.798	0.774	0.841	0.774	0.915	0.794	0.830
NAC	0.522	0.739	0.466	0.830	0.447	0.911	0.449	0.931	0.478	0.876
CUAC	0.682	0.654	0.699	0.773	0.684	0.766	0.831	0.846	0.677	0.782
TGRA	0.765	0.706	0.686	0.790	0.675	0.822	0.716	0.899	0.686	0.818

**Table 5:** Improvement in input–output indicators in the four major regions of the TGRA in 2020.

DMU	N1	N2	U1	U2	Y1	Z1
HRA	−0.163	−0.139	−0.137	−0.255	0.046	0.041
CEAC	−0.085	−0.076	−0.038	−0.034	0.077	0.114
NAC	−0.217	−0.159	−0.338	−0.325	0.067	−0.057
CUAC	−0.028	−0.123	−0.041	−0.034	0.167	−0.041
TGRA	−0.098	−0.115	−0.103	−0.116	0.099	0.025

Note: N1 and N2 represent per-capita education expenditure and per capita, science and technology expenditure, respectively; U1 and U2 represent resources consumption and environmental pollution, respectively; Y1 represents comprehensive well-being output indices; Z1 represents per-capita GDP.

**Table 6:** Influencing factors index system.

Category	Independent Variable	Variable Explanation	Data Type	References
Economic development level	X1 GDP per capita (CNY)	Regional Economic Strength Status	Statistics	[41,47,73]
	X2 Industrial value-added/GDP (%)	Industrial Structure	Statistics	[47,73]
	X3 Total import and export/GDP (%)	Openness Level	Statistics	[74]
Policy system	X4 Per-capita expenditure on science and technology (CNY)	Science and Technology Level	Statistics	[43]
	X5 Fiscal expenditure per capita (CNY)	Government Capability	Statistics	[41,73]
Social-living condition	X6 Number of health technicians per 1000 people (person)	Medical Level	Statistics	[75]
	X7 Urban population/total population (%)	Urbanization	Statistics	[41]
Natural environment	X8 NDVI	Vegetation Cover Level	Raster Data	[16]
	X9 Percentage of good days (%)	Air Quality Statistics	Statistics	[73]

**Table 7:** Geodetector results of EWP in the TGRA in 2010, 2014, 2017, and 2020.

Independent Variable	*p* Value	Significance	2010	2014	2017	2020
X1	0.000	0.01	0.530	0.505	0.668	0.584
X2	0.000	0.01	0.337	0.311	0.427	0.474
X3	0.000	0.01	0.192	0.143	0.264	0.260
X4	0.000	0.01	0.389	0.529	0.540	0.300
X5	0.000	0.01	0.159	0.162	0.111	0.282
X6	0.000	0.01	0.192	0.367	0.446	0.032
X7	0.000	0.01	0.334	0.382	0.638	0.645
X8	0.000	0.01	0.065	0.113	0.078	0.133
X9	0.000	0.01	0.335	0.337	0.176	0.245

## Data Availability

Not applicable.
